# Differences between therapeutic mechanisms of resmetirom and semaglutide against MASH in western diet-fed MC4R-knockout mice

**DOI:** 10.1038/s41598-025-24927-3

**Published:** 2025-11-20

**Authors:** Takumi Sugawara, Kosuke Hitaka, Mitsuharu Matsumoto, Sayuri Nakamura, Ryosuke Kobayashi, Hitoshi Kandori, Yasunori Nio

**Affiliations:** 1 Pharmacology Business Unit, Metabolic Syndrome Group, Axcelead Drug Discovery Partners, Inc, 26-1, Muraoka-Higashi 2- chome, Fujusawa, Kanagawa Japan; 2 Pharmacology Business Unit, Integrated Pathology Group, Axcelead Drug Discovery Partners, Inc, 26-1 Muraoka-Higashi 2-chome, Fujisawa, Kanagawa, Japan

**Keywords:** Metabolic dysfunction-associated steatotic liver disease, Melanocortin 4 receptor knockout, Resmetirom, Semaglutide, Fibrosis, Drug discovery, Diseases, Endocrinology, Gastroenterology, Medical research

## Abstract

Metabolic dysfunction-associated steatotic liver disease (MASLD) can progress to steatohepatitis (MASH), which is closely associated with obesity and insulin resistance. Resmetirom, and semaglutide, have been shown to have therapeutic effects in clinical studies. We compared these mechanisms in western diet (WD)-fed melanocortin 4 receptor knockout (MC4R-KO) mice, a human MASH pathology model. Male MC4R-KO mice were fed WD for 6 weeks starting from 22 weeks of age for disease induction and were administered drugs for 7 weeks with WD feeding, for a total duration of 13 weeks. Both resmetirom and semaglutide treatments for 7 weeks substantially improved these parameters. Although resmetirome and semaglutide improved liver hydroxyproline deposition and total fat mass, semaglutide markedly suppressed total lean mass. Moreover, resmetirom enhanced oxygen consumption, whereas semaglutide reduced energy expenditure. Histopathological evaluation showed that resmetirom significantly and semaglutide tended to improve liver steatosis score. On the fibrosis score, semaglutide significantly reduced it. Resmetirom and semaglutide have different mechanisms of action against MASH. Similar to clinical evidence, semaglutide treatment, might cause muscle mass reduction due to food intake suppression. This is the first study to simultaneously compare the effects of resmetirom and semaglutide on MASH phenotypes and reveal the differences on their mechanisms of action in WD-fed MC4R-KO mice.

**Supplementary Information:**

The online version contains supplementary material available at 10.1038/s41598-025-24927-3.

## Introduction

The prevalence of metabolic dysfunction-associated steatotic liver disease (MASLD) is rapidly increasing worldwide. It is now the most common liver disorder in the Western world, with an incidence rate of over 25%^1–4^. MASLD is strongly associated with metabolic abnormalities, such as obesity, insulin resistance, and type 2 diabetes mellitus, and encompasses complicated and extensive liver diseases, including asymptomatic steatosis and more aggressive metabolic dysfunction-associated steatohepatitis (MASH)^5,6^. MASH is characterized by steatosis, cytoskeletal damage (hepatocellular ballooning), lobular inflammation, and fibrosis^7^. MASH is considered progressive; the disease progresses to liver cirrhosis, then to hepatocellular carcinoma, and consequently, increases mortality rates^8,9^. Resmetirom, a thyroid hormone receptor (THR)-β agonist, has been FDA-approved for MASH treatment through its favorable efficacy, safety profiles^10^. This approval is a critical advancement in MASH therapy and provides a roadmap for additional therapies. The relationship between MASLD and TSH have been researched; hypothyroidism is considerably associated with the presence and severity of MASLD, and titrated levothyroxine dosing decreases hepatic lipid content^11,12^. However, systemic dosing of thyroid hormone has concerning adverse effects through THR-α expression in heart^13^. In contrast, THR-β is highly expressed in hepatocytes and regulates systemic lipid levels and metabolic pathways in the liver^14^. In addition, the expression of THR-β in the liver is inversely correlated with nonalcoholic fatty liver disease (NAFLD) activity score (NAS)^15^. Resmetirom has higher selectivity for THR-β than for THR-α and is preferentially taken up in the liver through the organic anion transporting polypeptide 1B1 receptor^16–18^. Resmetirom has shown ameliorative effects on liver fibrosis, steatosis, and inflammation in vivo using diet-induced obese (DIO) mice fed with diets such as Amylin liver non-alcoholic steatohepatitis (AMLN) diet and high fat diet (HFD)^14,19,20^. However, DIO and chemically induced mouse models do not fully reflect human MASH pathology. Although DIO mice were obese, the phenotypes of insulin resistance, hyperphagia, steatohepatitis, and fibrosis were either not observed or were mild^21^. For example, Lin et al.^22^ used a DIO mice model fed a high fat diet (60 kcal%) to evaluate the effect of THR-β agonists on metabolic function; however, its anti-fibrotic effect was not investigated. The DIO mouse model showed mild MASH pathology characterized by NAS, but did not exhibit liver inflammation or fibrosis.^22^ Nutrient-deficient diets low in or devoid of methionine and/or choline are used to induce severe liver fibrosis. In addition, chemically induced models, such as the CCl_4_-induced liver damage model, have been used to study the mechanisms of hepatic fibrosis progression. However, neither nutrient-deficient nor chemically induced models fully reflect human MASH pathology, as these models are not obese but show weight loss^23,24^. Melanocortin 4 receptor (MC4R) is a seven-transmembrane G-protein-coupled receptor expressed in the hypothalamic nuclei, and MC4R-knockout (KO) mice are implicated in the regulation of appetite, hyperphagia, and body weight^25^. Western diet (WD)-fed MC4R-KO mice and Gubra-Amylin non-alcoholic steatohepatitis (GAN) diet-fed mice models have been reported to highly resemble human MASH in terms of obesity, dyslipidemia, insulin resistance, liver injury, steatosis, and fibrosis^21,26,27^. However, the GAN diet-induced MASH model was established by feeding mice a GAN diet for approximately 30 weeks. In contrast, MC4R-KO mice showed a MASH-like pathology around 8 weeks of being fed a WD; therefore, WD-fed MC4R-KO mice are advantageous as a rodent MASH model owing to their ability to be established quickly. Therefore, combining WD with MC4R-KO mice, which exhibit substantial obesity and insulin resistance^21^, is an attractive MASH model with considerable fibrosis compared to the DIO mouse model. In this study, WD-fed MC4R-KO mice were generated, and the anti-MASH effects of resmetirom were confirmed. Moreover, the energy expenditure and fatty acid oxidation of resmetirom for anti-MASH effects were evaluated using the Oxymax system after repeated doses of resmetirom in WD-fed MC4R-KO mice.

Furthermore, single and dual agonists of glucagon-like peptide-1 (GLP-1) have ameliorative effects against MASH^28,29^. GLP-1 analogs were initially developed for type 2 diabetes mellitus, their efficacy against obesity was demonstrated, and they were used as anti-obesity drugs^30^. Moreover, a > 7% reduction in body weight improved NAS in patients with MASH^31,32^. Therefore, the therapeutic effect of GLP-1 analogs on MASH is attributed to body weight reduction through a decrease in appetite, followed by improvement in dyslipidemia and hepatic inflammation^33,34^. Accordingly, in this study, the anti-MASH effect of a GLP-1 analog was evaluated using WD-fed MC4R-KO mice, and its efficacy and scores were compared with that of resmetirom by simultaneous administration in the same study.

## Results

### MASH phenotypes of WD-fed MC4R-KO mice

The experimental protocol is illustrated in Supplementary Fig. 1. WD-fed MC4R-KO mice were generated after 6 weeks of feeding on WD at 22 weeks of age. The mice showed pronounced increases in body weight, food intake, and levels of liver injury markers, such as plasma alanine transaminase (ALT), aspartate aminotransferase (AST), tissue inhibitor of metalloproteinase-1 (TIMP-1), triglyceride (TG), cholesterol, and low-density lipoprotein cholesterol (LDL-C), compared to normal mice. In addition, WD-fed MC4R-KO mice showed a substantial increase in plasma insulin levels compared with those in normal mice (Supplementary Table 1). These changes in the parameters resemble those observed in patients with MASH.

### Effects of resmetirom and semaglutide on food intake, body weight, body composition, and tissue weight

**Fig. 1:**
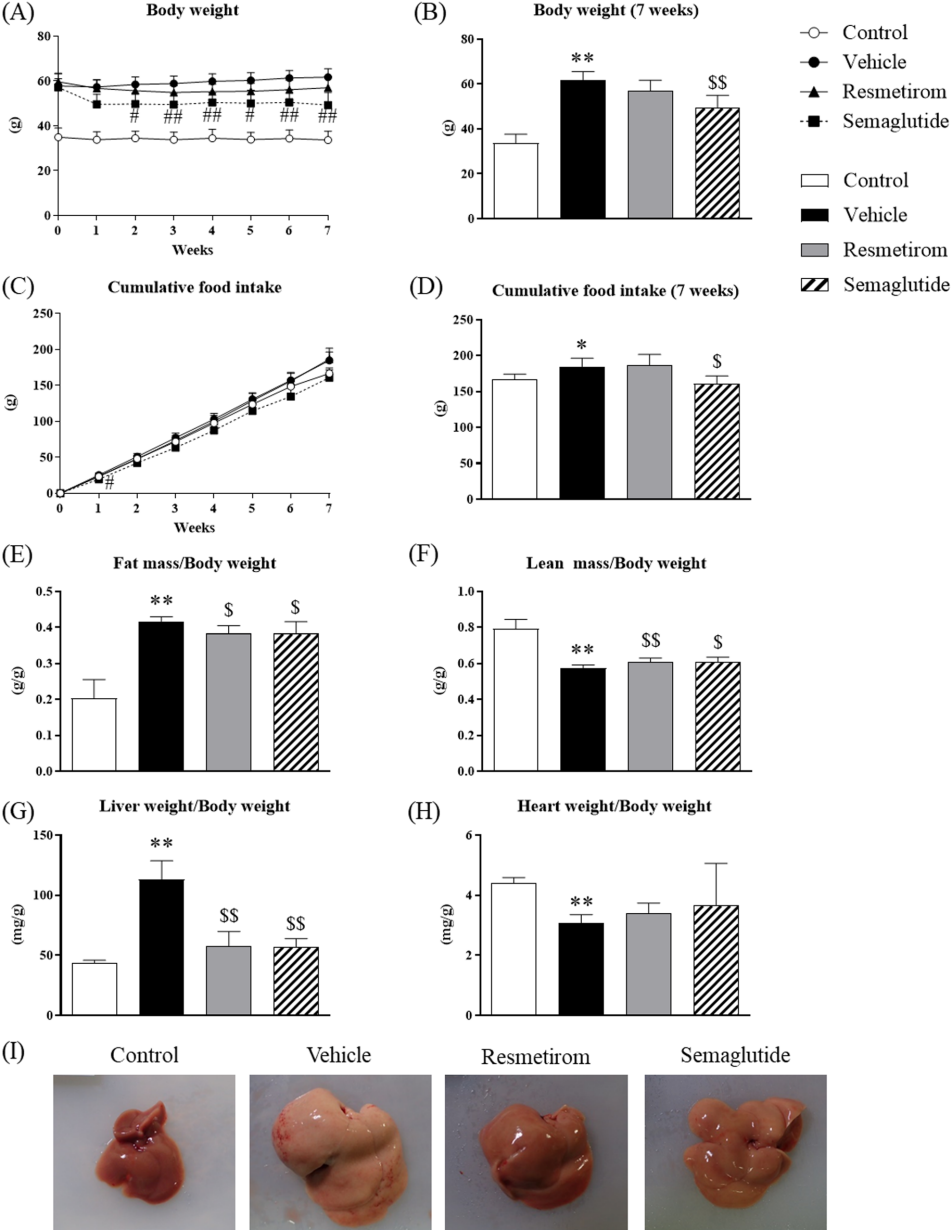
Effects of resmetirom and semaglutide on food intake, body weight, body composition, and tissue weight. (**A**) Body weight change for 7 weeks and (**B**) body weight after 7 weeks treatment. (**C**) Cumulative food intake for 7 weeks and (**D**) total cumulative food intake after 7 weeks treatment. (**E**) Fat mass and (**F**) lean mass relative to body weight determined via EchoMRI after 7 weeks treatment. (**G**) Liver and (**H**) heart weight relative to body weight. (**I**) Representative images of liver after treatment. Data are presented as the mean + standard deviation (SD). ^#^*p* < 0.05, ^##^*p* < 0.01, vs. vehicle (Dunnett’s test followed by Bonferroni correction). *p < 0.05, **p < 0.01, vs. control (Student’s t-test). ^$^*p* < 0.05, ^$$^*p* < 0.01, vs. vehicle (Dunnett’s test).

The effects of resmetirom and semaglutide on the food intake, body weight, and body composition of WD-fed MC4R-KO mice were investigated. Although semaglutide (Rybelsus) is orally bioavailable using the absorption enhancer salcaprozate sodium (SNAC) and is administered orally for diabetes, semaglutide (without SNAC) has been administered subcutaneously in clinical trials for patients with MASH. Therefore, in this study, we subcutaneously administered semaglutide to mice to assess its translational relevance in humans. Semaglutide markedly decreased body weight (Fig. 1A,B) and cumulative food intake compared to those in vehicle-treated mice (Fig. 1C,D); however, resmetirom treatment did not change body weight (Fig. 1A,B) or food intake (Fig. 1C,D) in WD-fed MC4R-KO mice. Body weight compositions of both fat and lean masses (Fig. 1E,F) were measured using an Echo-MRI system. Both resmetirom and semaglutide substantially decreased fat mass relative to body weight and total fat mass (Fig. 1E, Supplementary Fig. 2A). Regarding lean mass, semaglutide significantly suppressed total lean mass (Supplementary Fig. 2B) and slightly increased lean mass relative to body weight (Fig. 1F). Resmetirom also slightly increased lean mass relative to body weight (Fig. 1F) but there was no effect on total lean mass (Supplementary Fig. 2B). The liver weight of vehicle-treated WD-fed MC4R-KO mice was approximately three times that of control mice, and both resmetirom and semaglutide markedly reduced the liver weight (Fig. 1G, Supplementary Fig. 2C). After 7 weeks of resmetirom and semaglutide treatment in WD-fed MC4R-KO mice, no effect on the weight of the heart, which expresses THR-α, was observed (Fig. 1H, Supplementary Fig. 2D). In addition to liver enlargement, the livers of WD-fed MC4R-KO mice were pale and the liver surface had a granular texture (Fig. 1I). In contrast, the appearance of the drug-treated group was similar to that of the control group.

### Effect of resmetirom and semaglutide on liver injury markers and plasma LDL-C levels in WD-fed MC4R-KO mice

**Fig. 2:**
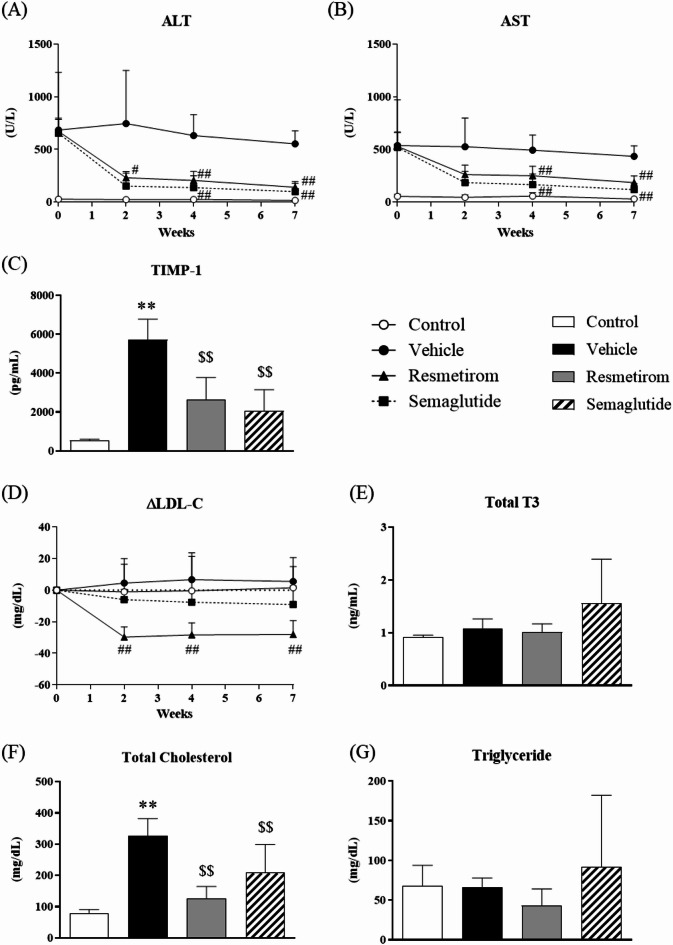
Effect of resmetirom and semaglutide on liver injury markers and plasma LDL-C levels in WD-fed MC4R-KO mice. (**A**) Time course of plasma ALT and (**B**) plasma AST levels for 7 weeks. (**C**) Plasma TIMP-1 concentration after 7 weeks treatment. (**D**) Time course of rate of plasma LDL-C concentration from the values before treatment for 7 weeks. (**E**) Plasma total T3 concentration. (**F**) Plasma total cholesterol and (**G**) triglyceride concentration after 7 weeks treatment. Data are represented as the mean + SD. LDL-C, low-density lipoprotein cholesterol; WD, western diet; MC4R, melanocortin 4 receptor; KO, knockout; ALT, alanine transaminase; AST, aspartate; TIMP-1, tissue inhibitor of metalloproteinase-1; SD, standard deviation. ^#^*p* < 0.05, ^##^*p* < 0.01, vs. vehicle (Dunnett’s test followed by Bonferroni correction). *p < 0.05, **p < 0.01, vs. control (Student’s t-test). ^$^*p* < 0.05, ^$$^*p* < 0.01, vs. vehicle (Dunnett’s test).

The effects of resmetirom and semaglutide on MASH were confirmed in the WD-fed MC4R-KO mice. Resmetirom and semaglutide treatments considerably reduced plasma ALT, AST, and TIMP-1 levels in WD-fed MC4R-KO mice, indicating a reduction in liver injury and fibrosis (Fig. 2A–C). Resmetirom showed a notable decrease of plasma LDL-C levels compared with those in vehicle-treated MC4R-KO mice (Fig. 2D). In contrast, the total plasma T3 levels were not influenced by resmetirom, indicating that resmetirom did not disrupt thyroid hormone homeostasis (Fig. 2E). In terms of other lipid parameters, total plasma cholesterol also increased in the WD-fed MC4R-KO mice and decreased in the drug-treated groups (Fig. 2F). There were no notable changes in TG levels in any group (Fig. 2G).

### Evaluation of liver hydroxyproline, TG, and total cholesterol (TC) content

**Fig. 3:**
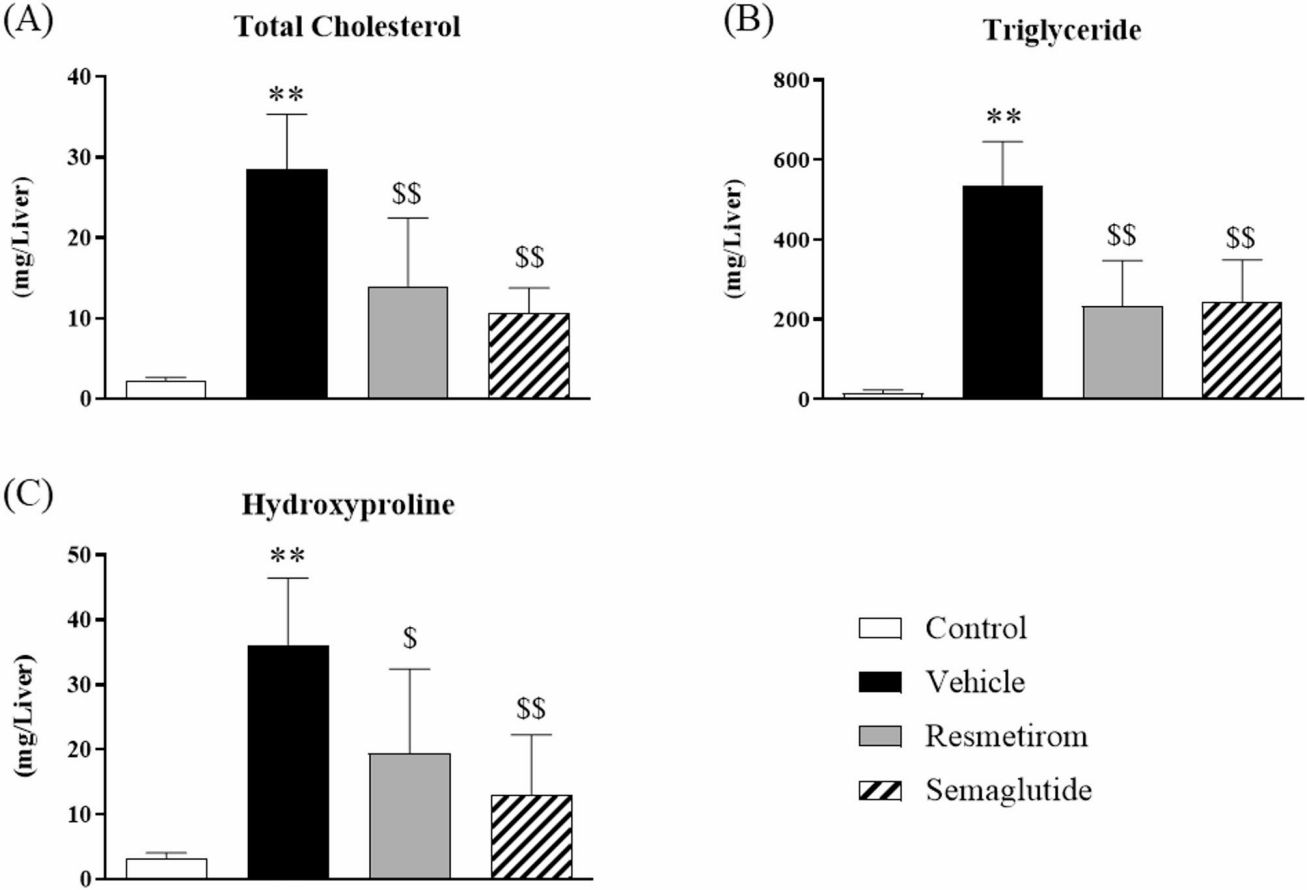
Evaluation of liver TG, TC, and hydroxyproline contents. (**A**) Total cholesterol, (**B**) triglyceride, and (**C**) hydroxyproline content in the liver after 7 weeks treatment. Data are presented as the mean + standard deviation (SD). **p < 0.01, vs. control (Student’s t-test). ^$^*p* < 0.05, ^$$^*p* < 0.01, vs. vehicle (Dunnett’s test).

Cholesterol, TG, and hydroxyproline levels were measured to confirm qualitative changes in the liver. Similar to the plasma cholesterol level, liver cholesterol increased in WD-fed MC4R-KO mice and was reduced by resmetirom and semaglutide treatment (Fig. 3A). Interestingly, the change in TG levels in the liver was similar to that of cholesterol levels (Fig. 3B), even though plasma TG levels were constant in all groups. Levels of hydroxyproline, a component of collagen used to assess tissue fibrosis, were higher in WD-fed MC4R-KO mice than in control mice. This suggests the development of liver fibrosis in the MASH mouse model. Both resmetirom and semaglutide markedly lowered the hepatic hydroxyproline content (Fig. 3C).

### Comparison of oxygen consumption, energy expenditure, and respiratory exchange ratio (RER) between resmetirom and semaglutide

**Fig. 4:**
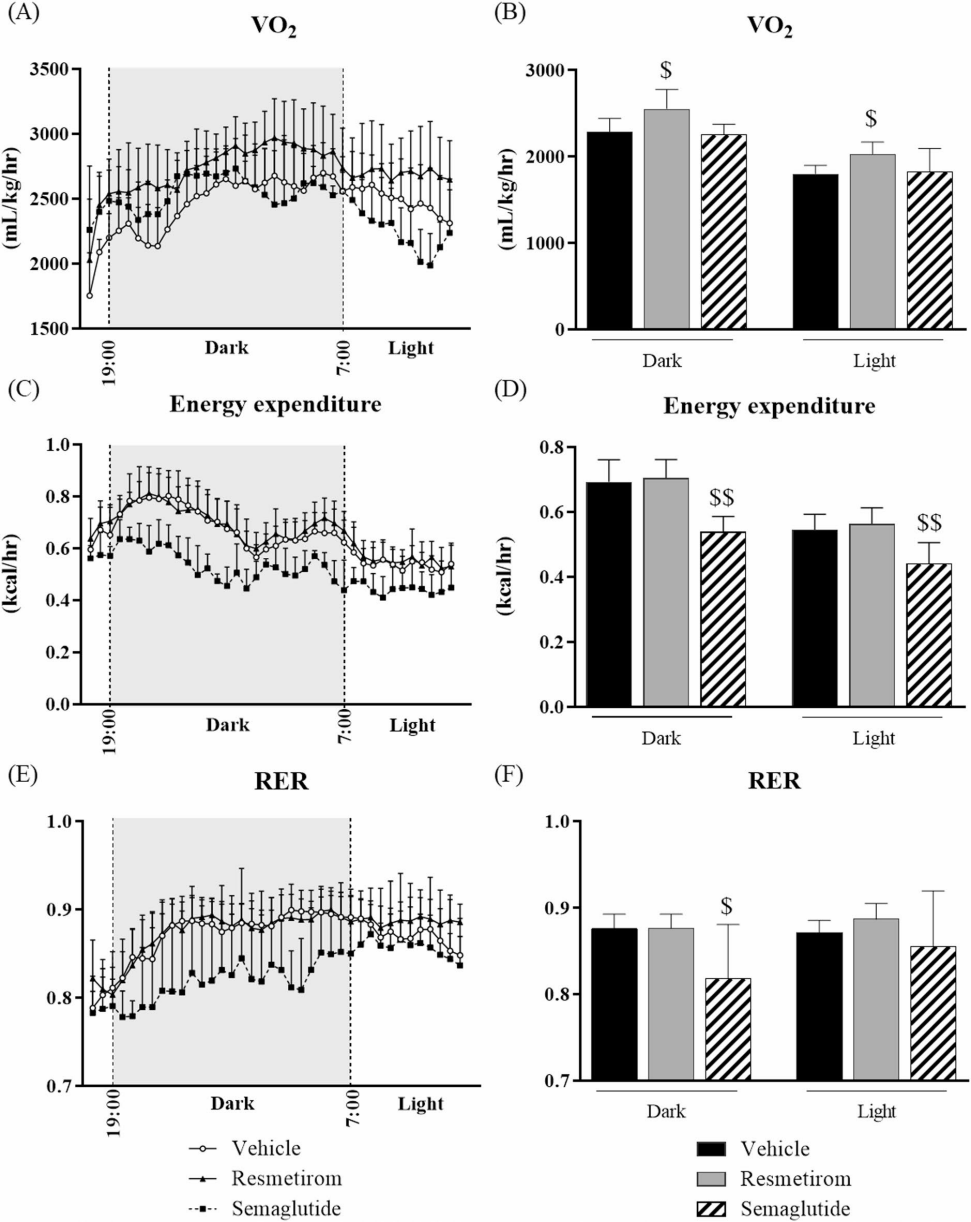
Comparison of oxygen consumption, energy expenditure, and RER between resmetirom and semaglutide. The time course of the metabolic rates was monitored continuously for 19 h using the Oxymax system. (**A**) oxygen consumption, (**C**) energy expenditure, and (**E**) RER. Shaded regions represent the dark phase of a 12-h light-dark cycle. (**B**) Average oxygen consumption, (**D**) energy expenditure, and (**F**) RER during the light and dark periods. Data are represented as the mean + SD. RER, respiratory exchange ratio; SD, standard deviation. ^$^*p* < 0.05, ^$$^*p* < 0.01, vs. vehicle (Dunnett’s test).

Activation of THR-β in the liver affects fatty acid metabolism and improves hepatic fat content. Therefore, in this study, the energy expenditure, oxygen consumption, and RER in WD-fed MC4R-KO mice treated with resmetirom and semaglutide were analyzed using the Oxymax system. Resmetirom substantially increased oxygen consumption (Fig. 4A,B) but did not change energy expenditure (Fig. 4C,D), whereas semaglutide considerably reduced energy expenditure and RER (Fig. 4D–F).

### Effect of gene expression after 7 weeks of resmetirom and semaglutide administration

Considering that the pathology of MASH is related to inflammation, fibrosis, and lipid metabolism, the expression of related genes was examined by Quantitative Real-Time Polymerase Chain Reaction (qRT-PCR). Among the inflammation-related genes, *Il1b* and *Ccl2* expression was markedly increased, whereas *Il6* expression tended to increase in the livers of WD-fed MC4R-KO mice. Resmetirom markedly suppressed *Ccl2* expression and tended to suppress *Il1b* and *Il6* expression. In contrast, semaglutide treatment resulted in a lower reduction in the expression of these genes (Table 1). The expression of fibrosis-related genes, such as *Col1a1*, *Col3a1*, and *Spp1*, was upregulated in WD-fed MC4R-KO mice, and both resmetirom and semaglutide tended to decrease the expression of these genes. Among the lipid metabolism-related genes, *Scd1*, *Fabp4*, and *Mogat2* were significantly upregulated in the WD-fed MC4R-KO mice. Resmetirom and semaglutide significantly suppressed *Mogat2* expression and tended to suppress *Scd1* and *Fabp4* expression.

**Table 1:** Effect of gene expression after 7 weeks of Resmetirom and semaglutide administration.

Mice	Control	MC4R KO		
Treatment	Vehicle	Vehicle	Resmetirom	Semaglutide
	(*n* = 4)	(*n* = 8)	(*n* = 8)	(*n* = 4)
Inflammation-related gene expression				
*Tnf*	1.00 ± 0.14	7.45 ± 3.89**	5.96 ± 3.91	5.91 ± 3.99
*Il1b*	1.00 ± 0.24	4.43 ± 1.75**	2.43 ± 0.88	3.66 ± 3.16
*Il6*	1.00 ± 0.40	3.78 ± 2.74	2.00 ± 1.51	2.45 ± 1.19
*Ccl2*	1.00 ± 0.18	16.54 ± 6.39**	7.54 ± 3.81^$$^	11.12 ± 5.34
Fibrosis-related gene expression				
*Acta2*	1.00 ± 0.13	2.66 ± 0.74**	2.33 ± 0.66	2.73 ± 1.31
*Col1a1*	1.00 ± 0.48	22.06 ± 12.86**	11.44 ± 10.15	11.14 ± 8.14
*Col3a1*	1.00 ± 0.31	9.58 ± 5.26**	5.38 ± 4.92	4.99 ± 2.84
*Spp1*	1.00 ± 0.26	10.84 ± 5.16**	5.45 ± 4.71	5.03 ± 3.35
Lipid metabolism-related gene expression				
*Scd1*	1.00 ± 0.41	2.13 ± 0.43**	1.73 ± 0.31	1.99 ± 0.74
*Fabp4*	1.00 ± 0.16	3.74 ± 1.16**	2.03 ± 0.79	4.43 ± 4.93
*Mogat2*	1.00 ± 0.23	2.65 ± 0.57**	0.85 ± 0.32^$$^	1.35 ± 0.73^$$^

Fold changes in the mRNA expression of inflammation-, fibrosis-, and lipid metabolism-related genes in the liver were normalized to those in the control group. Glyceraldehyde-3-phosphate dehydrogenase (*Gapdh*) was used as an endogenous control. Data are presented as mean ± standard deviation (SD). MC4R, melanocortin 4 receptor; KO, knockout; *Tnf*, tumor necrosis factor; *Il1b*, interleukin 1 beta; *Il6*, interleukin 6; *Ccl2*, C-C motif chemokine ligand 2; *Col1a1*, collagen type 1 alpha 1; *Col3a1*, collagen type 3 alpha 1; *Spp1*, osteopontin; *Scd1*, stearoyl-coenzyme A desaturase 1; *Fabp4*, fatty acid-binding protein 4; *Mogat2*, monoacylglycerol O-acyltransferase 2.

**p < 0.01, vs. control (Student’s t-test).

^$$^*p* < 0.01, vs. vehicle (Dunnett test).

### Histopathological evaluation for NAS and fibrosis

**Fig. 5:**
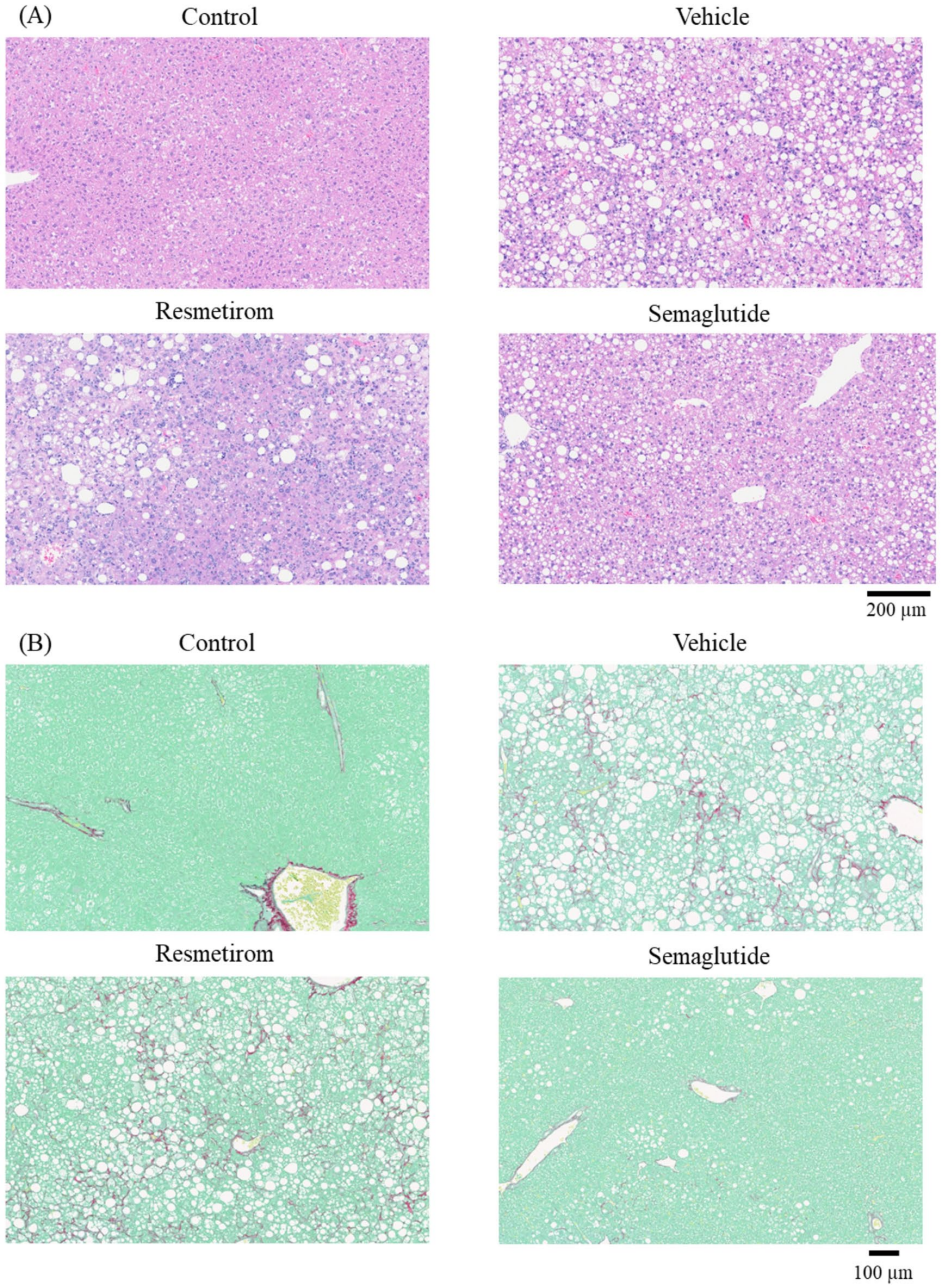
Histopathology for NAS and fibrosis. (**A**) Representative images of liver sections stained with hematoxylin & eosin. Large-sized lipid droplets were increased in the vehicle group compared to the control group, whereas amelioration was observed in both the resmetirom and semaglutide-treated groups. (**B**) Representative images of liver sections stained with Sirius Red. HE, hematoxylin & eosin.

Liver steatosis and lobular inflammation were evaluated using hematoxylin and eosin (HE)-stained specimens (Fig. 5A; Table 2). Vehicle-treated WD-fed MC4R-KO mice showed an increase in these scores and a prominent increase in liver steatosis. In terms of balloon degeneration of the NAS, these mice did not show any clear histopathological changes. These results indicated that WD-fed MC4R-KO mice exhibited severe steatosis with mild inflammation in the liver. Treatment with resmetirom and semaglutide considerably improved the liver steatosis score, suggesting that resmetirom and semaglutide decreased NAS compared to that with the vehicle treatment. In terms of the lobular inflammation score, no remarkable effects were observed with either resmetirom or semaglutide.

**Table 2:** Evaluation of NAFLD activity score and quantification of Sirius red-positive areas.

Mice	Control	MC4R KO		
Treatment	Vehicle	Vehicle	Resmetirom	Semaglutide
	(*n* = 4)	(*n* = 8)	(*n* = 8)	(*n* = 4)
Histopathology				
Steatosis	0.0 ± 0.0	3.0 ± 0.0**	1.8 ± 0.7^$$^	2.3 ± 1.0
Lobular inflammation	0.0 ± 0.0	1.0 ± 0.0**	1.0 ± 0.0	0.8 ± 0.5
NAFLD activity score	0.0 ± 0.0	4.0 ± 0.0**	2.8 ± 0.7^$$^	3.0 ± 1.4
Fibrosis analysis				
Fibrosis score	0.0 ± 0.0	1.0 ± 0.0**	1.1 ± 0.4	0.5 ± 0.6^$^
Sirius red-positive area (%)	0.24 ± 0.04	2.07 ± 0.91**	2.29 ± 1.37	1.20 ± 0.90

Individual scores for steatosis (0–3) and inflammation (0–2) were provided and added to determine the NAFLD activity score as a semi-quantitative measure of disease severity. Fibrosis was evaluated and expressed by score 0 to 4 as a semi-quantitative measure. Sirius red-positive areas were quantified using Halo AI. Data are presented as mean ± standard deviation (SD). MC4R, melanocortin 4 receptor; KO, knockout; NAFLD, nonalcoholic fatty liver disease.

**p < 0.01, vs. control (Student’s t-test).

^$^*p* < 0.05, ^$$^*p* < 0.01, vs. vehicle (Dunnett test).

The histological evaluation of liver fibrosis score and quantification of Sirius red-positive area were performed using Sirius red-stained specimens (Fig. 5B; Table 2). In accordance with elevated plasma TIMP-1 levels, the fibrosis score and Sirius red-positive area in the livers of WD-fed MC4R-KO mice were increased and broader than that in the control group. Despite the reduction in plasma TIMP-1, hepatic hydroxyproline, and the expression of fibrosis-related genes by resmetirom and semaglutide, there was no change in the fibrosis score and Sirius red-positive area with resmetirom. On the other hand, semaglutide significantly reduced fibrosis score.

## Discussion

Resmetirom was approved by the FDA as the first and thus far only MASH therapeutic drug, and the GLP-1 analog semaglutide showed positive effects in patients with MASH in a phase III clinical trial^35^. This study simultaneously evaluated the effects of resmetirom and semaglutide on WD-fed MC4R-KO mice. In this study, WD-fed MC4R-KO mice showed characteristics similar to those of human patients with MASH, such as increased ALT, AST, TIMP-1, TG, TC, and insulin levels (Supplementary Table 1). THR is highly expressed in the liver and the severity of MASLD is associated with reduced THR-β expression in the liver^15^. Moreover, THR-β in the liver regulates plasma LDL-C concentrations by tuning LDL receptor (LDLR) expression in the liver, and LDLR incorporates LDL into cells^36,37^. Many MASLD patients also have obesity, diabetes, and a risk of cardiovascular events; therefore, an LDL-lowering effect is preferable for MASH patients^38^. Furthermore, THR-β controls fat synthesis, regulates fatty acid oxidation and cholesterol metabolism, improves mitochondrial function, and reduces inflammation and fibrosis^39,40^. Accordingly, resmetirom, an approved drug for MASH treatment, decreases LDL-C levels and improves liver fibrosis^41^. A decrease in LDL-C levels was attributed to LDLR upregulation in the liver^36,37^. In this study, the effect of resmetirom on THR-β was confirmed by a decrease in plasma LDL-C levels of WD-fed MC4R-KO mice. In contrast, total plasma T3 was not affected by resmetirom, indicating that resmetirom did not disrupt thyroid hormone homeostasis.

MASLD is strongly associated with metabolic abnormalities such as obesity, and anti-obesity is thought to be a potential therapeutic strategy. GLP-1 analogs have been actively developed as candidates for MASH therapeutic drugs because of their role in reducing body weight^42^. Like resmetirom, GLP-1 analogs show indirect anti-inflammatory and anti-fibrotic effects by ameliorating metabolic abnormalities. The therapeutic effect of GLP-1 on MASH is thought to be due to a reduction in body weight caused by a decrease in appetite and insulin resistance, which leads to an improvement in dyslipidemia and hepatic inflammation^33,34^. Suitably, this study also showed that semaglutide reduced food intake and body weight.

As described above, although resmetirom and semaglutide have different mechanisms, they both improved liver injury, as reflected by a decline in ALT and AST levels and liver hydroxyproline content; however, some parameters differed in this study. Body mass analysis using an echo-MRI system showed that semaglutide reduced both total lean and total fat masses. Muscle and bone weights are included in lean mass. Loss of lean mass results in a reduction in energy expenditure and bone density, and skeletal muscle reduction is considered the main reason for body weight regain after discontinuation of semaglutide^43,44^.

In the present study, for the first time, a notable reduction in energy expenditure was observed in semaglutide-treated WD-fed MC4R-KO mice. These effects may be due to the reduction in lean mass, including muscle and bone mass. Therefore, semaglutide may cause muscle atrophy and osteoporosis. In contrast, resmetirom reduced fat mass, but not lean mass. Furthermore, resmetirom-treated mice exhibited increased oxygen consumption, which may have led to increased muscle mass. Therefore, combination therapy with resmetirom and semaglutide would be an ideal treatment with a synergistic effect on MASH and would reduce the risk of muscle mass reduction.

Similar to the plasma parameters reflecting MASH pathology, lipid accumulation in the liver, enhanced transcription of inflammation-related genes, and liver histopathology such as steatosis and lobular inflammation were also exacerbated in WD-fed MC4R-KO mice. However, lobular inflammation was weak and ballooning degeneration was not clearly observed in a previous study^45^. Although the NAS scoring method for humans includes ballooning degeneration, the evaluation of ballooning in the MASH mouse model is subject to observer variation, and disagreement exists even among experts^46^. Therefore, ballooning was not included in the NAS scoring in this study, but both resmetirom and semaglutide improved the NAS, mainly due to a reduction in the steatosis score. The reduction in the steatosis score was reflected in the decrease in liver TG and TC content and lipid metabolism-related gene expression.

Regarding fibrosis, WD-fed MC4R KO mice showed increased plasma TIMP-1 and liver hydroxyproline content, and an enhanced Sirius red-positive area, indicating fibrosis progression. However, histological evaluation of liver fibrosis did not show differences between vehicle and resmetirom, although these drugs improved the plasma and liver biochemical markers. Although semaglutide significantly suppressed fibrosis score, it did not significantly reduced Sirius red-positive area. Similar results were observed for the other MASH models. Nielsen et al.^47^ reported that interventional treatment with resmetirom and semaglutide did not result in a considerable reduction in the Sirius red-positive area in choline-deficient L-amino acid-defined high-fat diet (CDAA-HFD)-fed mice, although CDAA-HFD-fed mice showed a reduction in body weight. In GAN-diet-induced MASH model mice, resmetirom reduced steatosis and lobular inflammation, but did not improve the liver fibrosis score^48^. Kannt et al.^14^ reported that mice fed an AMLN diet for 34 weeks and administered resmetirom for 8 weeks did not show a decrease in the picrosirius staining fractional area and postulated that a longer treatment duration was needed. In contrast, Wang et al.^19^ reported that resmetirom treatment for 8 weeks after 25 weeks of feeding mice an AMLN diet improved liver fibrosis; therefore, the effect of resmetirom against liver fibrosis varies depending on the fibrosis level attributed to the duration of diet feeding and the type of MASH model. Longer treatment durations may be necessary to improve liver fibrosis in WD-fed MC4R-KO mice. Given that the phase 3 clinical trial of resmetirom also had a long duration of 52 weeks, it is possible that more repeated dosing times are needed to improve fibrosis in WD-fed MC4R-KO mice. In clinical point of view, similar to the results of this study, plasma biochemical parameters were considerably improved in the resmetirom-treated group in a phase 3 clinical trial^41^. However, the percentage of patients with improved fibrosis without worsening NAFLD was approximately 25% after 52 weeks of treatment. Taken together, the anti-fibrotic effect of resmetirom is not very strong, and resmetirom indirectly improves fibrosis by improving hepatic steatosis and suppressing inflammation. Regarding anti-fibrotic effect, the ameliorative effect of semaglutide would be derived from appetite suppression and body weight reduction, which improve fat accumulation in the liver. In contrast, resmetirom did not reduce body weight and lean mass. Resmetirom also increased oxygen consumption and improved liver steatosis.

**Fig. 6:**
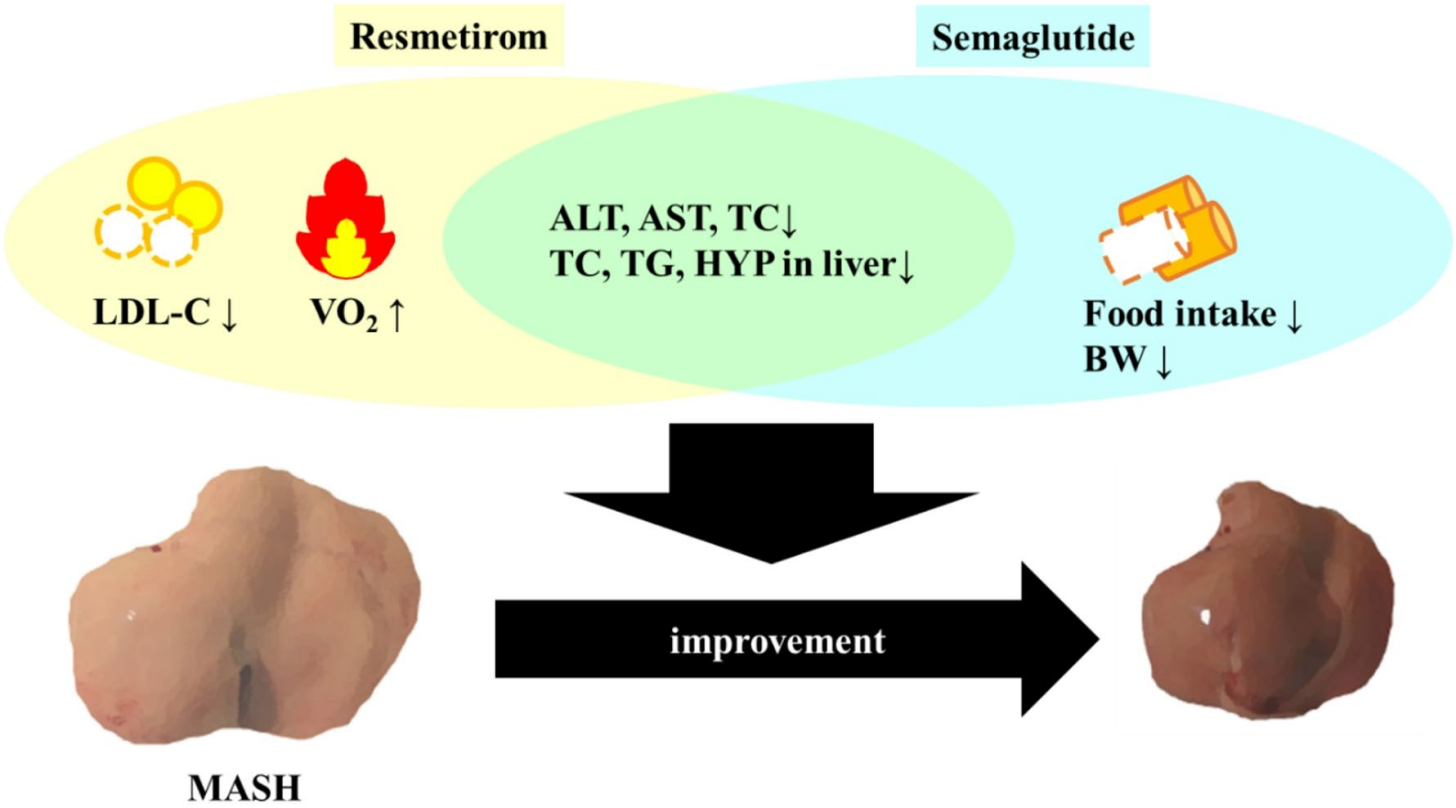
Schema of the amelioration effects of resmetirom and semaglutide. LDL-C, low-density lipoprotein cholesterol; VO_2_, oxygen consumption; ALT, alanine transaminase; AST, aspartate aminotransferase; TC, total cholesterol; HYP, hydroxyproline; BW, body weight; MASH, metabolic dysfunction-associated steatohepatitis.

In this study, we revealed the different mechanisms of the anti-MASH effects of resmetirom and semaglutide (Fig. 6) but there are some limitations. First, there are differences in administration rout: resmetirom was orally administered and semaglutide was subcutaneously injected. Oral gavages are stressful, and there are likely differences in the uptake and metabolism between compounds administered orally (p.o.) and subcutaneously (s.c.), such as stimulation of gut hormones or altered first-pass metabolism. Therefore, the possibility that differences in the treatment methods affect certain parameters should be considered.

Second, regarding the control group, chow-fed C57BL/6 mice are not the best controls for this study.

Littermate wild type mice for MC4R-KO mice would be the best control for comparison. Also, it would be preferable to have an MC4R-KO mouse group with a chow diet to observe the difference in disease progression compared to the WD-fed mice. In this study, however, the objective was to evaluate the efficacy of drugs against MASH. Therefore, we considered chow-fed C57BL/6 mice, which are non-pathological, to be sufficient controls since WD-fed MC4R-KO mice are a well-established MASH model that shows both steatosis and fibrosis^27^. Finally, the semaglutide group was under-powered (*n* = 4). Therefore, some parameters did not show a statistically significant difference with semaglutide but exhibited a tendency toward improvement. Further studies with a larger number of animals are necessary to determine significant ameliorative effects of semaglutide on MASH treatment. Although there are these limitations, these results suggest that the possibility of combination therapy with resmetirom and semaglutide, may be beneficial for MASH treatment and improve MASH treatment outcomes.

## Methods

### Compounds

Resmetirom and semaglutide were purchased from MedChemExpress (HY-12216, HY-114118; MedChemExpress, USA). Methyl cellulose solution was purchased from Fujifilm Wako Pure Chemical Industries.

### Animals

Mc4r knockout (KO) mice were generated by introducing CRISPR/Cas9 ribonucleoprotein (RNP) complexes into oocytes from C57BL/6J mice (CLEA Japan, Inc.). Guide RNAs (gRNAs) were designed to target the 5′ and 3′ untranslated regions (UTRs) of the Mc4r gene. The gRNA sequences were as follows: 5′-gaggttggatcagttcaagg-3′, 5′-caggaggttggatcagttca-3′, 5′-ggaagtacactgctcagatc-3′, and 5′-gggaagtacactgctcagat-3′. Genotyping of F0 mice was performed by quantitative PCR (qPCR) to determine the Mc4r copy number. The primers and probes used for Mc4r and the internal control Ngf were as follows (5′–3′): -Mc4r Forward: atttgcagcctgctttcca, Reverse: tggagcgcgtaaaagatagtga FAM-MGB Probe: tgcggtggacaggta, -Ngf, Forward: tgcatagcgtaatgtccatgttg, Reverse: tctccttctgggacattgctatc, VIC-TAMRA Probe: acggttctgcctgtacgccgatca (all primers and probes were synthesized by Thermo Fisher Scientific Inc.). The Mc4r-deleted allele was confirmed by Sanger sequencing. Male mice lacking the entire Mc4r coding region were fertilized with oocytes from female C57BL/6J mice by in vitro fertilization (IVF) to generate heterozygous offspring of both sexes, which were subsequently intercrossed to obtain homozygous knockout mice. Male MC4R-KO mice were fed WD (D12079B; Research Diets, New Brunswick, Canada) for 6 weeks starting from 22 weeks of age for disease induction and were administered drugs for 7 weeks with WD feeding. The total duration of the WD feeding was 13 weeks. Normal chow-fed wild-type male C57BL/6J mice of the same age were used as lean controls. Both groups had ad libitum access to food and water. The mice were individually housed under controlled temperature and humidity and a 12-h light-dark cycle (lights on 7:00–19:00). All animal experiments were approved by the Institutional Animal Care and Use Committee of Shonan Research Center (AU-00040846). All animal experiments were performed in accordance with relevant guidelines and regulations. The study was conducted in accordance with the ARRIVE guidelines.

### Repeated dosing study of resmetirom and semaglutide efficacy in WD-fed MC4R-KO mice

WD-fed MC4R-KO mice were divided into the following three groups: vehicle (0.5% methyl cellulose [MC], p.o.) (*n* = 8), resmetirom (5 mg/kg, p.o.) (*n* = 8), semaglutide (0.1 mg/kg s.c.) (*n* = 4). These groups were based on plasma levels of ALT, AST, and LDL-C levels and body weight. Age-matched normal CE-2-fed wild-type mice were administered the vehicle via oral gavage (0.5% MC, p.o.) (*n* = 4). MC (0.5%) and resmetirom were orally administered and semaglutide was subcutaneously injected once daily for 7 weeks. The body weight and food intake were measured weekly. After 2 and 4 weeks of treatment, plasma parameters were measured. After 7 weeks of treatment, all mice were anesthetized with isoflurane (3–5%), and after it was confirmed that the animal was in a deep anesthetized state based on the pain reflex, all blood was collected from the abdominal vena cava, and the animal was euthanized by exsanguination. Livers were harvested for histopathological and gene expression analyses in the same manner. All animal experiments were conducted with the approval of the Institutional Animal Care and Use Committee and the Shonan Health Innovation Park.

### Analysis of plasma and liver biochemistry

Blood was collected from the tail vein before treatment and at 2 and 4 weeks after treatment or from the inferior vena cava at 7 weeks after treatment in the fed state. Plasma ALT, AST, LDL-C, TG, and TC levels were measured enzymatically using the Clinical Analyzer 7180 (Hitachi High-Technologies, Tokyo, Japan). The plasma tissue inhibitor of metalloproteinase-1 (TIMP-1) concentration was measured using a mouse TIMP-1 Quantikine enzyme-linked immunosorbent assay (ELISA) kit (R&D Systems, Minneapolis, USA). Plasma insulin concentrations were measured using an ultrasensitive mouse insulin ELISA kit (Morinaga Institute of Biological Science, Kanagawa, Japan). The total plasma T3 concentration was measured using a Total Triiodothyronine ELISA KIT (Alpha Diagnostic International, USA). To measure hepatic TGs and cholesterol, aliquots of the liver were homogenized at a concentration of 100 mg of tissue per 1 mL of saline, and the homogenate was mixed thoroughly with a mixture of hexane and 2-propanol (3:2). After centrifugation, lipid-containing upper organic layers were collected. Hexane and 2-propanol solutions were added and the upper layer was collected again. The collected upper layers were dried and the residue was dissolved in 2-propanol. TG and cholesterol concentrations were measured using the TG and cholesterol E tests, respectively (Fujifilm Wako Pure Chemical Industries, Osaka, Japan). Hepatic hydroxyproline content was measured using a commercially available Total Collagen Kit (Quickzyme Biosciences, Leiden, Netherlands) according to the manufacturer’s instructions.

### Measurement of body composition

Fat and lean mass compositions were measured 7 weeks after treatment using a quantitative magnetic resonance method without anesthesia, according to the manufacturer’s instructions (EchoMRI-900; Hitachi Aloka Medical Ltd., Tokyo, Japan).

### Analysis of energy expenditure via oxymax

WD-fed MC4R-KO mice treated with the compounds for 7 weeks were housed individually in the metabolic chamber of an Oxymax system (Columbus Instructions, Columbus, OH, USA) according to the manufacturer’s instructions. At 5:00 p.m., the mice were administered vehicle (0.5% MC solution), resmetirom, or semaglutide, and their metabolic rates and respiratory quotients were measured from 6 p.m. to 1 p.m. (19:00–7:00, dark phase; 7:00–19:00, light phase).

### Gene expression analysis via qRT-PCR

Total RNA was isolated from 50 to 100 mg of liver tissue using the RNeasy Mini kit (Qiagen, Tokyo, Japan), followed by reverse transcription using a high-capacity RNA-to-cDNA kit (Thermo Fisher Scientific, Tokyo, Japan) according to the manufacturer’s instructions. cDNA was amplified using TaqMan Universal Master Mix II (Invitrogen, Tokyo, Japan) and ABI7900 (Life Technologies, Tokyo, Japan) according to the manufacturer’s instructions. Commercially available primer-probe sets were used (Applied Biosystems, Waltham, MA, USA). The sets of qRT-PCR probes were as follows: tumor necrosis factor (*Tnf*; Mm00443260), interleukin 1 beta (*Il1b*; Mm00434228), interleukin 6 (*Il6*; Mm00446190), C-C motif chemokine ligand 2 (*Ccl2*; Mm00441242), collagen type 1 alpha 1 (*Col1a1*; Mm00801666), collagen type 3 alpha 1 (*Col3a1*; Mm00802300), osteopontin (*Spp1*; Mm00436767), stearoyl-Coenzyme A desaturase 1 (*Scd1*; Mm00772290), monoacylglycerol O-acyltransferase 2 (*Mogat2*; Mm00624192), and fatty acid binding protein 4 (*Fabp4*; Mm00445878). Glyceraldehyde-3-phosphate dehydrogenase (*Gapdh*; Mm99999915) was used as an endogenous control gene, and relative mRNA expression was calculated via the ΔΔCt method.

### Histopathological analysis

Dissected liver tissues were fixed in 10% neutral formalin and embedded in paraffin. Paraffin section (3 μm) were stained with HE, and NAS was determined by pathologists^46,49^. The steatosis score assessed the quantity of large lipid droplets, but not foamy micro vesicles, from 0 to 3 (0: <5%; 1:5%–33%; 2:34%–66%; 3: >67%). The fibrosis score was defined from 0 to 4 levels with these criteria (0, none, 1, perisinusoidal zone 3 or portal fibrosis, 2, perisinusoidal and periportal fibrosis without bridging, 3, bridging fibrosis, 4, cirrhosis). Lobular inflammation was defined as the presence of two or more inflammatory cells within the lobule. Foci were counted at 20× magnification (0, none; 1, 2 foci per 20×; 2, > 2 foci per 20×). Ballooning was evaluated in all animals but was not observed in this study. For fibrosis evaluation, paraffin section (3 μm) were stained with 0.1% Sirius red and 0.1% Fast Green FCF solutions. Whole-slide digital images were acquired using a NanoZoomer S60 (Hamamatsu Photonics, Shizuoka, Japan). Detection of Sirius Red-positive area was performed using Halo^®^ Area Quantification analysis software version v2.3.1 combined with DenseNet classifier of Halo AI v4.0 (plugin) (Indica Labs, Albuquerque, NM, USA). First, the region of interest (ROI) was set by detecting the hepatic parenchyma, excluding connective tissues of the capsules and around large blood vessels, using the DenseNet classifier. Next, the fibrotic area was detected using Area Quantification software based on Sirius Red staining, and the percentage of Sirius Red-positive areas in the total ROIs was evaluated.

### Statistical analysis

All data are represented as mean + standard deviation. To confirm the establishment of the disease state, statistical differences between normal CE-2-fed wild-type mice and vehicle-treated WD-fed MC4R-KO mice were analyzed using Student’s t-test. To evaluate the effects of the drugs, the statistical differences between the vehicle and drug treatment groups were analyzed using Dunnett’s test. The Bonferroni correction was used to compare multiple time points. Statistical significance was set at *P* < 0.05.

## Supplementary Information

Below is the link to the electronic supplementary material.

Supplementary Material 1

Supplementary Material 2

## Data Availability

The data supporting the findings of this study are included in this article. The raw data generated and/or analyzed during the current study are available from the corresponding author upon reasonable request.

## Abbreviations

MASLD: Metabolic dysfunction-associated steatotic liver disease
MASH: Metabolic dysfunction-associated steatohepatitis
WD: Western diet
MC4R: Melanocortin 4 receptor
KO: Knockout
THR: Thyroid hormone receptor
NAFLD: Nonalcoholic fatty liver disease
NAS: Nonalcoholic fatty liver disease activity score
AMLN: Amylin liver non-alcoholic steatohepatitis
HFD: High fat diet
DIO: Diet-induced obese
GAN: Gubra-Amylin non-alcoholic steatohepatitis
GLP-1: Glucagon-like peptide-1
MC: Methyl cellulose
p.o.: Per os
ALT: Alanine transaminase
AST: Aspartate aminotransferase
LDL-C: Low-density lipoprotein cholesterol
TG: Triglyceride
TC: Total cholesterol
TIMP-1: Tissue inhibitor of metalloproteinase-1
ELISA: Enzyme-linked immunosorbent assay
qRT-PCR: Quantitative real-time polymerase chain reaction
Tnf: Tumor necrosis factor
Il1b: Interleukin 1 beta
Il6: Interleukin 6
Ccl2: C-C motif chemokine ligand 2
Col1a1: Collagen type 1 alpha 1
Col3a1: Collagen type 3 alpha 1
Spp1: Osteopontin
Scd1: Stearoyl-Coenzyme A desaturase 1
Fabp4: Fatty acid binding protein 4
Mogat2: Monoacylglycerol O-acyltransferase 2
Gapdh: Glyceraldehyde-3-phosphate dehydrogenase
HE: Hematoxylin and eosin
ROI: Regions of interest
RER: Respiratory exchange ratio
LDLR: LDL receptor
CDAA-HFD: Choline-deficient L-amino acid defined high fat diet
