# Supplementary material for: Differences between therapeutic mechanisms of resmetirom and semaglutide against MASH in western diet-fed MC4R-knockout mice
Source: Sci Rep. 2025 Nov 20;15:41068. doi: 10.1038/s41598-025-24927-3 (PMC12635150; doi:10.1038/s41598-025-24927-3)
Supplement: Supplementary file 1 — Supplementary Material 1 [file 41598_2025_24927_MOESM1_ESM.docx]

**Supplementary Figure 1**

**
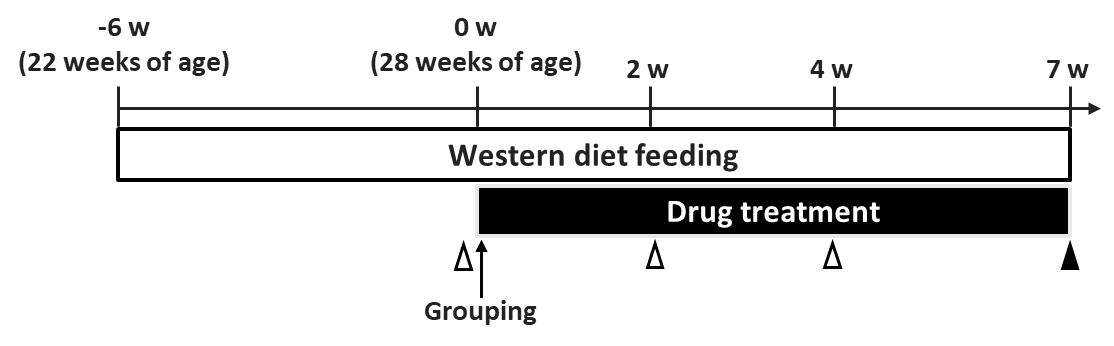
**

**Supplementary Figure 1. Experimental protocol.**

Experimental protocol for repeated dosing study using MC4R KO mice. 22 weeks of age male MC4R KO mice were fed with WD for 13 weeks. Drug treatments were started 6 weeks after pre-feeding. Drugs were administered for 7 weeks starting at 28 weeks of age. The white triangle indicates the collection of blood. The black triangle indicates the endpoint of this study.

**Supplementary Figure 2**

**
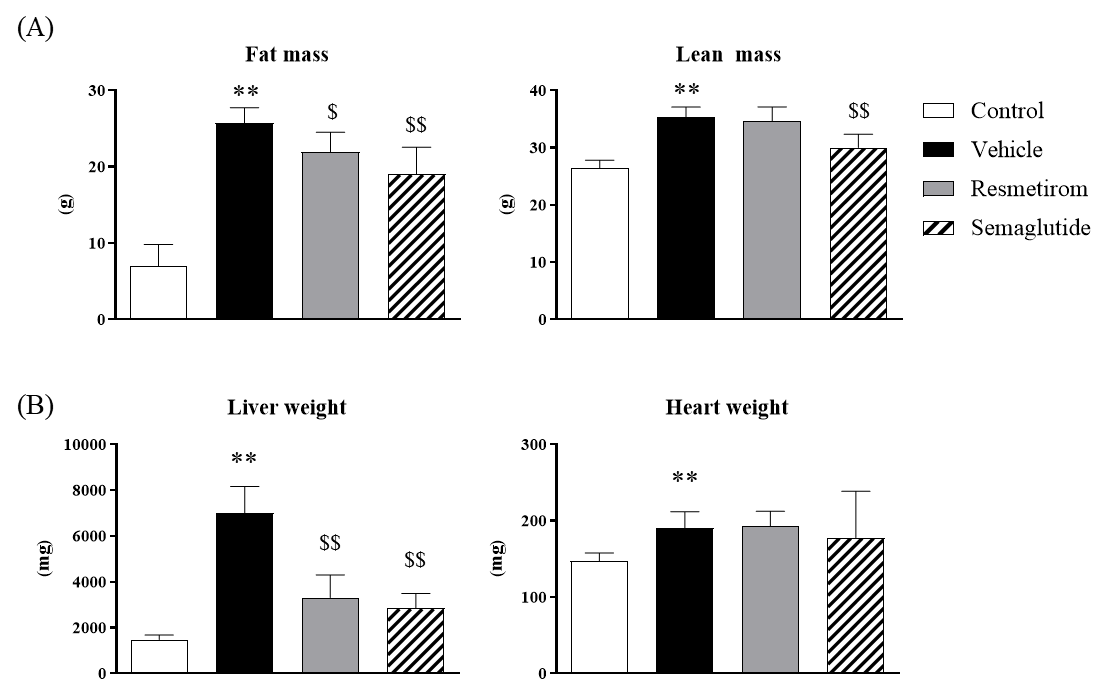
**

**Supplementary Figure 2. Effects of resmetirom and semaglutide on body composition, and tissue weight.**

(A) Fat mass and lean mass determined via EchoMRI at week 7. (D) Liver and heart weight. Data are presented as the mean + standard deviation (SD).

** p < 0.01, vs Control (Student’s t-test)

$ p < 0.05, $$ p < 0.01, vs Vehicle (Dunnett’s test)
