# Supplementary material for: Differences between therapeutic mechanisms of resmetirom and semaglutide against MASH in western diet-fed MC4R-knockout mice
Source: Sci Rep. 2025 Nov 20;15:41068. doi: 10.1038/s41598-025-24927-3 (PMC12635150; doi:10.1038/s41598-025-24927-3)
Supplement: Supplementary file 2 — Supplementary Material 2 [file 41598_2025_24927_MOESM2_ESM.docx]

**Supplementary Table 1. MASH phenotypes of WD-fed MC4R-KO mice.**

|  | Control | MC4R KO |
| --- | --- | --- |
| Body weight (g) | 34.1 ± 3.3 | 58.5 ± 3.9** |
| Food intake (g/day) | 3.56 ± 0.26 | 3.88 ± 0.30* |
| ALT (U/L) | 28.7 ± 6.9 | 680.9 ± 230.2^$$^ |
| AST (U/L) | 59.2 ± 25.4 | 531.7 ± 193.9^$$^ |
| TIMP-1 (pg/mL) | 1318.0 ± 187.1 | 6780.7 ± 2395.4^$$^ |
| Triglyceride (mg/dL) | 110.3 ± 51.5 | 76.1 ± 24.7 |
| Cholesterol (mg/dL) | 87.6 ± 9.8 | 322.4 ± 71.7^$$^ |
| LDL-C (mg/dL) | 6.7 ± 0.7 | 46.3 ± 10.6^$$^ |
| Insulin (ng/mL) | 2.1 ± 1.1 | 30.1 ± 23.7^$$^ |

MASH, metabolic dysfunction-associated steatohepatitis; WD, western diet; MC4R, melanocortin 4 receptor; KO, knockout; ALT, alanine transaminase; AST, aspartate aminotransferase; TIMP-1, tissue inhibitor of metalloproteinase-1; LDL-C, low-density lipoprotein cholesterol.

Plasma parameters were measured in lean control mice and WD fed-MC4R-KO mice after 6 weeks of WD feeding. Data are presented as the mean ± standard deviation (SD).

* p < 0.05, ** p < 0.01, vs Control (Student’s t-test)

$ p < 0.05, $$ p < 0.01, vs Control (Wilcoxon test)
